# Late pulmonary metastases of renal cell carcinoma immediately after post-transplantation immunosuppressive treatment: a case report

**DOI:** 10.1186/1752-1947-2-111

**Published:** 2008-04-18

**Authors:** Jose Manuel Cozar, Natalia Aptsiauri, Miguel Tallada, Federico Garrido, Francisco Ruiz-Cabello

**Affiliations:** 1 Servicio de Urología, Hospital Universitario Virgen de las Nieves, Granada, Spain; 2 Servicio de Análisis Clínicos, Hospital Universitario Virgen de las Nieves, Granada, Spain

## Abstract

**Introduction:**

We report a case of pulmonary metastatic recurrence of renal adenocarcinoma soon after radical nephrectomy that was followed by renal transplant and immunosuppressive medication. Increased risk of metastatic recurrence of renal cell carcinoma should be considered in the immediate post-transplant period when immunosuppressive medication is administered, even if nephrectomy had been performed many years earlier.

**Case presentation:**

In 1986 the patient demonstrated renal insufficiency secondary to mesangial glomerulonephritis. In 1992 he underwent left side radical nephrectomy with histopathological diagnosis of clear cell adenocarcinoma. Mesangial glomerulonephritis in the remaining right kidney progressed to end-stage renal failure. In October 2000 he received a kidney transplant from a cadaver and commenced immunosuppressive medication. Two months later, several nodules were found in his lungs, which were identified as metastases from the primary renal tumor that had been removed with the diseased kidney 8 years earlier.

**Conclusion:**

Recurrence of renal cell carcinoma metastases points to tumor dormancy and reflects a misbalance between effective tumor immune surveillance and immune escape. This case demonstrates that a state of tumor dormancy can be interrupted soon after administration of immunosuppressant medication.

## Introduction

Renal cell carcinoma (RCC) is the most common renal malignancy [1]. Late recurrence of metastatic renal carcinoma has previously been described [2-5]. The greatest risk of recurrence for RCC occurs within the first 5 years after nephrectomy [6]. Although, recurrences have been reported as late as 30 years following nephrectomy, rates of 43% in the first year, 70% within the second year, 80% within 3 years, and 93% within 5 years have been reported [7,8]. After nephrectomy, the incidence of RCC recurrence has been reported to be 7% with a median time of 38 months for T1 tumors, 26% with a median time of 32 months for T2 disease, and 39% with a median time to recurrence of 17 months for T3 tumors [9]. RCC has been shown to metastasize to almost all soft tissues in the body, but most commonly to the lung, followed by bone, liver, brain, and local recurrence [10]. RCC metastases occur frequently in the lung, affecting 3% to 16% of patients after nephrectomy [8,9,11].

It is well known that patients who have undergone solid organ transplantation and are taking immune suppressing drugs to prevent organ rejection are at a higher risk of getting cancer [12]. The most common malignancies are lymphoproliferative disorders (early after transplantation) and skin carcinomas (later after transplantation). In kidney transplant recipients, renal cell carcinoma (RCC) may occur either in the native kidney or, less frequently, in the grafted kidney [13,14]. High risk of metastatic recurrence in immunosupressed patients is often associated with interruption of tumor dormancy and proliferation of residual tumor cells that had survived the anti-tumor immune reactivity because of low immunogenicity [15,16]. Cancer patients with tumor dormancy can have an apparent balance between tumor cell replication and cell death, reflecting a balance between effective immune surveillance and immune escape [17].

Here we report a case of late and aggressive pulmonary metastatic progression of renal cancer in an immunosupressed patient soon after renal transplantation and many years after the initial removal of a malignant kidney. We believe that immunosuppressive medication provoked rapid metastatic recurrence in this patient.

## Case presentation

A 43-year-old male patient with a previous history of arterial hypertension and cholecystectomy for gallstone disease, presented in our clinic in 1986 with renal insufficiency secondary to mesangial glomerulonephritis and without any evidence of a renal mass as analyzed by sonography. In 1992, a renal mass on the left side, compatible with renal adenocarcinoma, was detected during a surveillance annual abdominal sonography and was later confirmed by CT scan. The diameter of the lesion was 5 × 5 cm. The same year, the patient underwent left side radical nephrectomy with histopathological diagnosis of clear cell adenocarcinoma pT2pN0M0 (primary tumor localized in the kidney parenchyma without metastases in the regional lymph nodes and without distant metastases according to "tumour-node-metastasis" (TNM) classification. Mesangial glomerulonephritis in the remaining right kidney progressed to end-stage renal failure. In 2000, the patient had peritoneal dialysis and, because thoracoabdominal CT examination revealed no signs of local or distant renal tumor recurrence, the patient was recommended for renal transplantation.

In October 2000, he received a renal transplant from cadaver and started immunosuppressive medication. The regimen included: Prednisolone, Tacrolimus (calcineurin inhibitor) and Mycophenolate. The immunosupression was not augmented since there were no signs of rejection.

**Figure 1:**
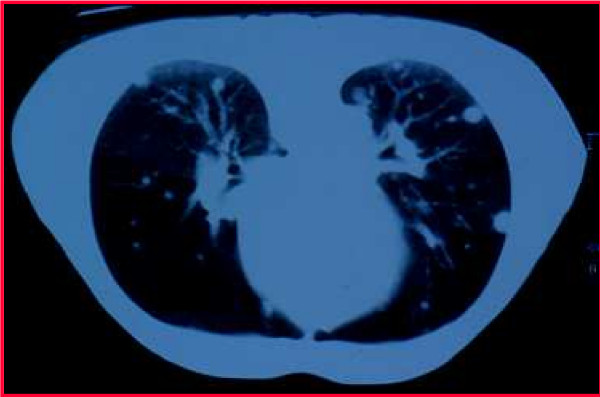
CT scan demonstrating pulmonary nodules.

**Figure 2:**
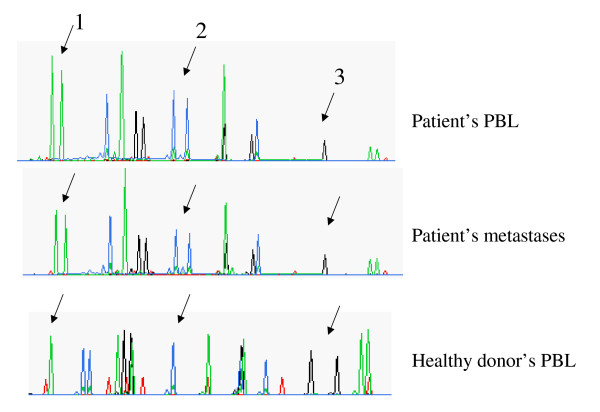
**Microsatellite analysis showing similar profile between metastases and peripheral blood lymphocytes (PBL)s of the patient**. This assay is commonly used for linkage mapping studies, association studies, and identification of organisms. It has been reported to be useful for assessment of chimerism in graft-versus-host disease, for identification of the site of origin of unknown primary tumor, and for determination of donor-recipient origin in posttransplant lymphoproliferative disorders. Microsatellites are short runs of tandemly repeated DNA that represent a primary source of human genetic diversity. The variations in these repeats are used for genetic identification purposes. It is based on PCR amplification of various microsatellite loci (or markers) using fluorescently labeled forward and unlabeled reverse primers. The PCR amplicons are separated by size and the labeled products are identified using fluorescence detectors. In this case the DNA was extracted from pulmonary metastases, patients PBLs, and from healthy donor PBLs. Arrows indicate differences in the microsatellite markers used: 1 – Amelogenin locus in X/Y chromosomes (patient and metastases have XY profile (male), donor has XX profile (female)); 2 – locus vWA in chromosome 12p (heterozygous in patient's PBLs and metastases, homozygous in donor PBLs); 3 – D7S820 marker in chromosome 7q (homozygous in patient's BPLs and metastases, heterozygous in donor PBLs).

In December of the same year, the patient presented with spontaneous pneumothorax on the left side and underwent pleural punction and aspiration. CT scan showed disseminated pulmonary nodules (Figure 1) with mediastinal adenopathy. Abdominal CT scan showed a functional transplanted right kidney without malignant lesions. There were no signs of tumor recurrence in the kidneys. Bone scintigraphy was negative for metastases. Histological analysis of biopsies of pleural parietal and pulmonary nodules from the left lung identified them as metastases from clear cell renal carcinoma. DNA microsatellite analyses using AmpFlSTR Profiler Plus PCR Amplification Kit (PE Applied Biosystems, Madrid, Spain) (Figure 2) demonstrated that the metastatic lesions did not originate from the donor kidney but from the the primary renal tumor removed with the diseased kidney 8 years earlier. Importantly, these metastatic nodules appeared two months after initiation of immunosuppressive treatment. It was decided to remove the transplant and stop the immunosuppressive medication. The patient was commenced on hemodialysis. In February 2001, immunotherapy with IL-2 and IFN-alpha was initiated, which was well tolerated by the patient. In August 2001, chest CT demonstrated enlargement and progression of the pulmonary metastases. The patient presented with asthenia, anorexia, anemia and bone pain, and he was referred to the Pain Care Unit. In October 2001, one year after commencement of immunosuppressive medication, the patient died.

## Discussion

It is well documented that metastatic recurrence is a frequent cause of cancer development even many years after removal of primary tumor [6]. It is also well known that immunosuppressive therapy in post-transplantation patients frequently induces various *de novo *cancers [12]. We believe that in our case tumor dormancy of residual malignant cells that lasted for 8 years, had been interrupted after the changes in the patient's immune status immediately following kidney transplantation and immunosuppressive regime. Persistence of circulating tumor cells may represent residual disease and is known to be associated with a higher risk of recurrence; however, the status of tumors during the period of dormancy is poorly understood. Various factors have been identified as possible contributors to tumor dormancy and subsequent recurrence, including tumor angiogenesis, cell proliferation and cell cycle arrest, cancer cell interactions with the microenvironment, and changes in immune status [18]. Various published results have described that oncogene inactivation can induce tumor regression and that oncogene reactivation leads to rapid tumor formation. There are reports demonstrating that at least in some cases tumor dormancy can be interrupted by a transient change in the microenvironment due to local inflammation. Animal studies also indicate the importance of T-cell immunity in the induction and maintenance of tumor dormancy. Dormant micrometastases may be triggered to expand when host or cancer cell factors change to promote progressive metastatic growth. In our case the immunosupression was a probable trigger of such expansion. Tumor dormancy represents an existence of an apparent balance between tumor cell elimination and proliferation, or between effective immune surveillance and immune escape. According to the theory of immunosurveillance, originally proposed by Burnet and Thomas in 1957, the immune system protects the host against tumor development by recognizing and eliminating transformed cells, therefore, the dysfunction of the immune system leads to increased risk of cancer incidence. Published data obtained from more recent mouse models and from human clinical trials supports this theory and suggests the existence of a more complex picture of immunosurveillance, which includes an active role of the immune system also in facilitating immunoselection of malignant cells and the emergence of tumor escape variants with low immunogenicity. New data indicate that the immune system can facilitate tumor progression, at least in part, by sculpturing the immunogenic phenotypes of tumors as they develop [19]. It has been confirmed in animal studies that tumors developing in immunodeficient mouse strains tend to be more immunogenic, while tumor cells arising in the presence of a fully functional immune system are less immunogenic. Thus, in the case of a functional immune system highly immunogenic tumor cells that are recognized by the immune system are eliminated while tumor variants that have better survival features, such as low immunogenicity, are selected. This selective pressure leads to the expansion of a new population of cells with multiple defects capable of evading immune response. These residual tumor cells may be circulating in patients in long remissions and they may represent a different stage of tumor control mechanisms, called an immunological equilibrium. Some of the immune escape mechanisms, notably downregulation of MHC class I molecules that are necessary for antigen presentation of tumor specific peptides to cytotoxic T-lymphocytes, are most pronounced in metastatic lesions, as it has been demonstrated in RCC [20]. This suggests that the selection pressure is strong in the metastatic stage of cancer development. Thus, the immune system might play a dual role: protecting the host by eliminating strong variants of tumor cells and promoting tumor development by selection of immunologically weak tumor variants that give rise to a new metastatic progression. Therefore, the immune system can maintain a subclinical tumor in an equilibrium state. We propose that a stage of immunological equilibrium between tumor growth and elimination had been reached in our patient after the excision of the malignant kidney. After transplantation, the administration of immunosuppressants changed the immunological equilibrium and probably triggered the pulmonary metastatic malignant growth of dormant tumor cells from the primary renal carcinoma. This supports the importance of immune surveillance as part of tumor-host interaction during cancer progression and the key role of tumor escape in immunosupressed individuals.

## Conclusion

In conclusion, this case demonstrates that dormant metastases can develop in immunocompromised cancer patients soon after administration of immunosuppressive medication even after a long cancer-free and metastasis-free period following removal of the primary tumor.

## Competing interests

The authors declare that they have no competing interests.

## Authors' contributions

JMC and MT are urologists who treated the patient for many years. NA, FG and FRC are immunologists who performed microsatellite analysis, analyzed the data and drafted the manuscript. All authors read and approved the final manuscript.

## Consent

Written informed patient consent was obtained for publication of this case report and accompanying images. A copy of the written consent is available for review by the Editor-in Chief of this journal.
